# The Association between Influenza Vaccination and COVID-19 and Its Outcomes: A Systematic Review and Meta-Analysis of Observational Studies

**DOI:** 10.3390/vaccines9050529

**Published:** 2021-05-20

**Authors:** Ruitong Wang, Min Liu, Jue Liu

**Affiliations:** 1 Department of Epidemiology and Biostatistics, School of Public Health, Peking University, Beijing 100191, China; wangruitong@pku.edu.cn (R.W.); liumin@bjmu.edu.cn (M.L.); 2 Institute for Global Health and Development, Peking University, Beijing 100191, China; 3 Key Laboratory of Reproductive Health, National Health Commission of the People’s Republic of China, Beijing 100083, China

**Keywords:** influenza vaccination, COVID-19, infection, outcome, meta-analysis

## Abstract

Influenza could circulate in parallel with COVID-19. In the context of COVID-19, some studies observed inverse associations between influenza vaccination and SARS-CoV-2 infection and clinical outcomes, while others did not. We conducted a meta-analysis to assess the association between influenza vaccination and SARS-CoV-2 infection and clinical outcomes, aiming to provide evidence for COVID-19 prevention and vaccination promotion. We searched four databases from inception to 10 March, 2021. Random effects and fixed effects models were used to pool odds ratios (ORs) and adjusted estimates with 95% confidence intervals (CIs). We used funnel plots to evaluate the publication bias, I2 statistics to evaluate the heterogeneity, and conducted subgroup analyses. Sixteen observational studies involving 290,327 participants were included. Influenza vaccination was associated with a lower risk of SARS-CoV-2 infection (pooled adjusted OR: 0.86, 95%CI: 0.81–0.91), while not significantly associated with adverse outcomes (intensive care: adjusted OR 0.63, 95%CI: 0.22–1.81; hospitalization: adjusted OR 0.74, 95%CI: 0.51–1.06; mortality: adjusted OR 0.89, 95%CI: 0.73–1.09). Our findings suggest that influenza vaccination is associated with a lower risk of SARS-CoV-2 infection. It is crucial for policy makers to implement strategies on influenza vaccination, for it may also have benefits for COVID-19 prevention.

## 1. Introduction

The coronavirus disease (COVID-19) is an acute respiratory infectious disease that was declared a global public health emergency by the World Health Organization (WHO) in January 2020 [1]. The global pandemic has hitherto caused 119 million cases of infection and 2 million cases of death [1], and imposed tremendous burden on global health and worldwide economics. Thus, effective cures and vaccines are imperatively needed to curtail the pandemic and decrease mortality. Seasonal influenza occurs from fall to spring annually, characterized by the circulation of influenza A or B virus [2]. Influenza and its complications could lead to increased worldwide mortality and morbidity, which remain a public health threat.

Due to the seasonality of influenza outbreaks and the continuous prevalence of COVID-19, influenza could circulate in parallel with COVID-19, which largely increases the potential risk of co-infection. Though little is known about the epidemiology and clinical outcomes of co-infection, extant literature has found that the co-infection with influenza A virus enhances the infectivity of COVID-19 in a broad range of cell types [3], whereas co-infected patients seem to present similar clinical symptoms and radiological images compared with patients infected with COVID-19 alone [4,5]. In the context of the COVID-19 pandemic, the dual infection of influenza and COVID-19 could bring extra burden to health care services by utilizing limited medical resources, increasing the difficulty of treatment and the uncertainty of prognosis. Annual influenza vaccination has long been recommended by WHO to prevent influenza, especially to the high-risk populations with disproportionate infection and severe complications, such as older adults (aged > 65 years) and pregnant women [6]. To date, no highly effective pharmaceutical treatment is available against COVID-19 [7]. Though COVID-19 vaccines remain the most effective long-term solution to combat COVID-19 pandemic [8], the overall effectiveness and safety of the licensed COVID-19 vaccines remain to be fully evaluated based on real-world evidence.

According to a previous study by Wolff [9], which investigated influenza vaccine-related virus interference by specific respiratory viruses (e.g., coronavirus, human bocavirus, and adenovirus), there was an increased odd of coronavirus in individuals receiving influenza vaccination. This finding raised much concerns of the possible relationship between influenza vaccination and coronavirus, especially in the COVID-19 pandemic. In addition, as COVID-19 and influenza are both respiratory infectious diseases caused by enveloped RNA viruses that share similarities in transmission routes and clinical characteristics [10], more and more researchers began to seek for relationships between SARS-CoV-2 infection and influenza immunity. Based on the assumptions, Del Riccio et al. [11] conducted a systematic review and found that there was overall no evidence to suggest a negative impact of influenza vaccination on SARS-CoV-2 related infections, illness, or deaths, while some of the included studies even reported significantly inverse associations. Though some of the recent studies have found that influenza vaccine uptake was negatively associated with COVID-19 incidence [12,13], severity [13,14], and mortality [13,15], others showed no evidence of such associations [16,17,18]. Therefore, a systematic review and meta-analysis of the association between influenza vaccination and SARS-CoV-2 infection and its outcomes is needed to provide conclusive evidence.

In the dual epidemics of COVID-19 and influenza, influenza vaccination has a more significant implication than ever for preventing both influenza and COVID-19. It is especially of great necessity for vulnerable populations to receive influenza vaccination. Given the limited data of COVID-19 vaccine effectiveness among vulnerable groups, as well as the necessity of influenza vaccination in the context of COVID-19, and the lack of conclusive evidence of influenza vaccination’s effect on SARS-CoV-2 infection and its clinical outcomes, there is a need to systematically assess the potential association between influenza vaccination and COVID-19. In this regard, we conducted a systematic review and meta-analysis to assess the overall association between influenza vaccination and SARS-CoV-2 infection and clinical outcomes, aiming to provide evidence for public health decision makers to develop COVID-19 preventive measures and provide implications for vaccination promotion.

## 2. Materials and Methods

### 2.1. Data Sources and Search Strategy

We searched PubMed, Embase, Web of Science, and Cochrane Library for eligible studies published from June 1971 to 10 March, 2021 using the following search terms: (‘flu’ OR ‘influenza’) AND (‘COVID-19’ OR ‘SARS-CoV-2’ OR ‘coronavirus’) AND (‘vaccination’ OR ‘vaccine’). We used EndNote 20 software to manage records, exclude duplicates, and screen abstracts and titles. This study was conducted in accordance with the Preferred Reporting Items for Systematic Reviews and Meta-Analyses (PRISMA) guidelines and the Meta-analysis of Observational Studies in Epidemiology (MOOSE) guidelines. We prospectively submitted the systematic review protocol for registration on PROSPERO (registration ID: CRD42021244442).

### 2.2. Data Sources and Search Strategy

We basically included observational studies that examined the association between influenza vaccination and SARS-CoV-2 infection or reported the association between influenza vaccination and clinical outcomes among SARS-CoV-2 infected populations. The following studies were excluded: (1) irrelevant to the subject of the meta-analysis, such as studies that did not use influenza vaccination as the exposure or did not report the outcomes; (2) insufficient data to calculate the odds ratio (OR) or select the adjusted estimates (aRR or aOR) on the association between influenza vaccination and SARS-CoV-2 infection and outcomes; (3) duplicate studies or overlapping participants; (4) reviews, editorials, conference papers, case reports or animal experiments; (5) studies that did not mention the identification of COVID-19. For example, the confirmed diagnosis of COVID-19 via reverse-transcription polymerase chain reaction (rt-PCR) test, serologic test, or other means were not mentioned in the text; and (6) studies that did not clarify the ascertainment of influenza vaccination (health system record/self-report).

Studies were identified by two investigators (R.W. and J.L.) independently following the criteria above, while discrepancies were resolved with a third investigator (M.L.).

### 2.3. Quality Assessment

We evaluated the risk of bias using the Newcastle–Ottawa quality assessment scale for cohort studies and case-control studies [19], while the methodological quality of cross-sectional studies was assessed using the checklist recommended by Agency for Healthcare Research and Quality (AHRQ) [20]. Cohort studies and case-control studies were classified as having low (≥7 stars), moderate (5–6 stars), and high (≤4 stars) risk of bias with an overall quality score of 9 stars. For cross-sectional studies, we assigned each item of the AHRQ checklist a score of 1 (answered “yes”) or 0 (answered “no” or “unclear”), and summarized scores across items to generate an overall quality score that ranged from 0 to 11. Low, moderate, and high risk of bias were identified as having a score of 8–11, 4–7 and 0–3, respectively. Two investigators (R.W. and J.L.) independently assessed study quality, with disagreements resolved by a third investigator (M.L.).

### 2.4. Data Extraction

The primary outcome was the association between influenza vaccination and SARS-CoV-2 infection, and the secondary outcome was the association between influenza vaccination and clinical outcomes of SARS-CoV-2 infection. The following data were extracted independently from the selected studies: (1) basic information of the studies, including first author, publication year, variables adjusted in the analysis and study design; (2) characteristics of the study population, including sample sizes, age groups, or regions; (3) seasons for influenza vaccination of the exposed group; (4) primary outcomes: the number of SARS-CoV-2 infected and non-infected people and by vaccination status (vaccinated or unvaccinated); (5) secondary outcomes: the number of influenza vaccinated and unvaccinated people and by clinical outcomes of SARS-CoV-2 infection (e.g., hospitalization, mortality, and intensive care); and (6) adjusted estimates (aRR or aOR) with 95% CI that were relevant to the primary or secondary outcomes. If a study reported the crude estimates or adjusted estimates of different influenza seasons, only the estimates of the most recent influenza season were included.

### 2.5. Data Synthesis and Statistical Analysis

We performed a meta-analysis to pool data from observational studies and assessed the overall associations between influenza vaccination and COVID-19 by clinical outcomes (infected vs. uninfected, hospitalization vs. non-hospitalization, death vs. alive, intensive care vs. non-intensive care). Random effects and fixed effects models were used to pool the crude ORs and adjusted ORs across studies separately. The pooled estimates were deemed significant when the according 95%CIs did not pass through zero and the *p* value was less than 0.05.

We conducted subgroup analyses to investigate the possible sources of heterogeneity by using study designs, sample sizes, and regions as grouping variables. We used the Q test to conduct subgroup comparisons and variables were considered significant between subgroups if the subgroup difference *p* value was less than 0.05. Sensitivity analyses were performed by omitting one study at a time to assess studies with notable impact and examine the robustness of the overall effect. Funnel plots and Egger’s tests were used to assess publication bias. We analyzed data using Stata version 16.0 and R version 4.0.2.

## 3. Results

### 3.1. Study Selection and Study Characteristics

**Figure 1:**
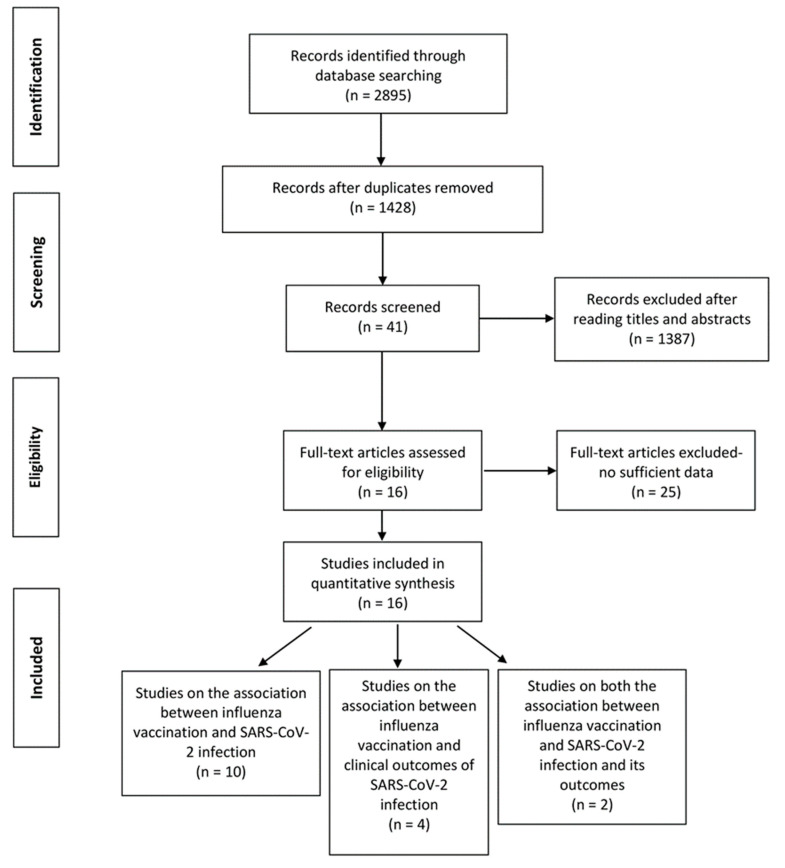
PRISMA flow diagram of the study selection procedure.

A total of 2895 records were retrieved from the four databases and 1467 duplicates were excluded. After screening titles and abstracts, we excluded 1387 reviews, conference papers, animal experiments, case reports, and other studies irrelevant to the subject or published before December 2019. Among the 41 articles assessed based on full texts, 25 articles were excluded for lacking specific data or did not meet the inclusion criteria. A total of 16 studies were finally included in the review (12 studies on the association between influenza vaccination and SARS-CoV-2 infection [16,18,21,22,23,24,25,26,27,28,29,30], 6 on the association between influenza vaccination and COVID-19 clinical outcomes [10,17,22,26,31,32], 2 studies containing data on both the associations [22,26]. Nine of the 12 studies on the association between influenza vaccination and SARS-CoV-2 infection contained adjusted estimates. One [21] of the 16 studies has moderate risk of bias, while the others have low risk of bias. The primary outcome (the association between influenza vaccination and SARS-CoV-2 infection) comprised a total of 208,132 people (72,820 vaccinated and 135,112 unvaccinated). The secondary outcome (the association between influenza vaccination and clinical outcomes of SARS-CoV-2 infection) comprised a total of 82,684 COVID-19 patients and was assessed by different outcomes (mortality, intensive care, and hospitalization). The study selection procedure is shown in Figure 1. The baseline characteristics of the included studies are listed in Table 1 (primary outcome) and Table 2 (secondary outcome). The adjusted variables of the included studies were basically age, sex, comorbidities, prescribed medications and smoking status, but were not the same across studies (see Table 1 and Table 2).

### 3.2. The Association between Influenza Vaccination and COVID-19 and Its Outcomes

**Figure 2:**
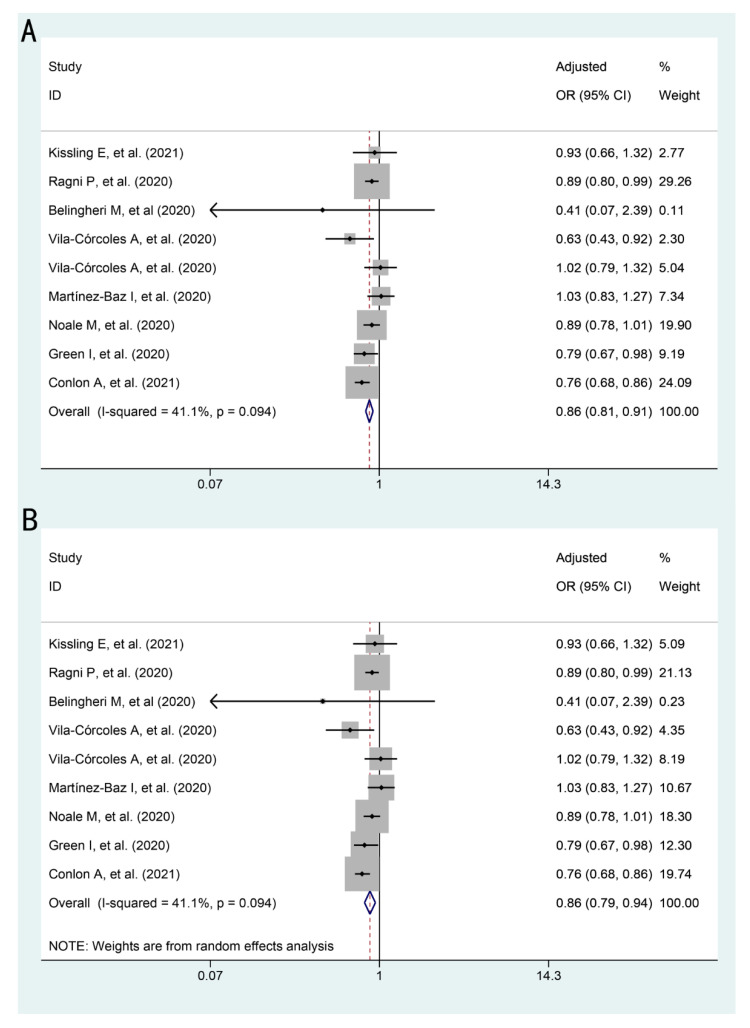
Forest plots for the association between influenza vaccination and SARS-CoV-2 infection: (**A**) adjusted OR by fixed effects model (**B**) adjusted OR by random effects model.

The association between influenza vaccination and SARS-CoV-2 infection is presented in Figure 2, Table 3 and Supplementary Table S1. Influenza vaccination was shown to be associated with a lower risk of SARS-CoV-2 infection in both models (fixed effects model: pooled adjusted OR: 0.86, 95%CI: 0.81–0.91; random effects model: pooled adjusted OR: 0.86, 95%CI: 0.79–0.94).

The association between influenza vaccination and COVID-19 outcomes are presented in Table 3 and Supplementary Table S1. The association between influenza vaccination and intensive care (adjusted OR: 0.63, 95%CI: 0.22–1.81), hospitalization (adjusted OR: 0.74, 95%CI: 0.51–1.06), or mortality (adjusted OR: 0.89, 95%CI: 0.73–1.09) among COVID-19 patients was not statistically significant by random effects model, while results by fixed effects model was somehow significant. This may be due to the substantial heterogeneity between the small number of studies (2–3 studies) and participants involved in each outcome.

### 3.3. Subgroup Analysis

**Figure 3:**
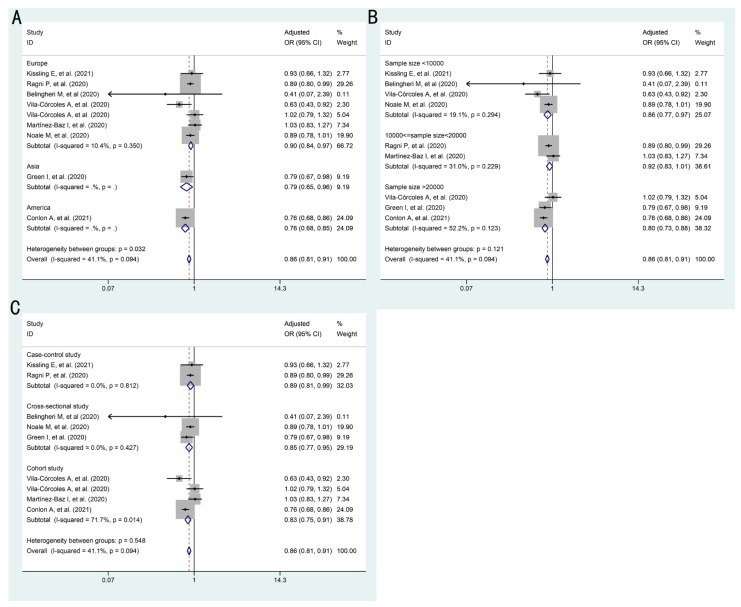
Forest plots for subgroup analysis on the association between influenza vaccination and SARS-CoV-2 infection by fixed effects model: (**A**) stratified by region (**B**) stratified by sample size (**C**) stratified by study design.

Given the heterogeneity among the outcomes of the association between influenza vaccination and SARS-CoV-2 infection (Figure 2, Table 3), we conducted subgroup analyses by sample sizes, regions, and study designs. The results of subgroup analyses are presented in Figure 3, Supplementary Figure S1, Table 4 and Supplementary Table S2. There was no significant evidence of association stratified by sample sizes or study designs (all *p* > 0.05). However, there was substantial heterogeneity between the adjusted estimates of studies in Europe (0.90, 95%CI: 0.84–0.97), America (0.76, 95%CI: 0.68–0.85), and Asia (0.79, 95%CI: 0.65–0.96, *p* = 0.03).

Owing to the paucity of the included studies on the association between influenza vaccination and any of the COVID-19 outcomes (mortality, hospitalization, and intensive care), planned subgroup analyses were not able to be performed.

### 3.4. Publication Bias and Sensitivity Analysis

In the sensitivity analyses regarding the association between influenza vaccination and SARS-CoV-2 infection, the pooled estimates were consistent when any one of the studies was omitted, which demonstrated the robustness of the results. Sensitivity analyses on the association between influenza vaccination and clinical outcomes among COVID-19 patients were not carried out owing to the sparseness of the included studies.

**Figure 4:**
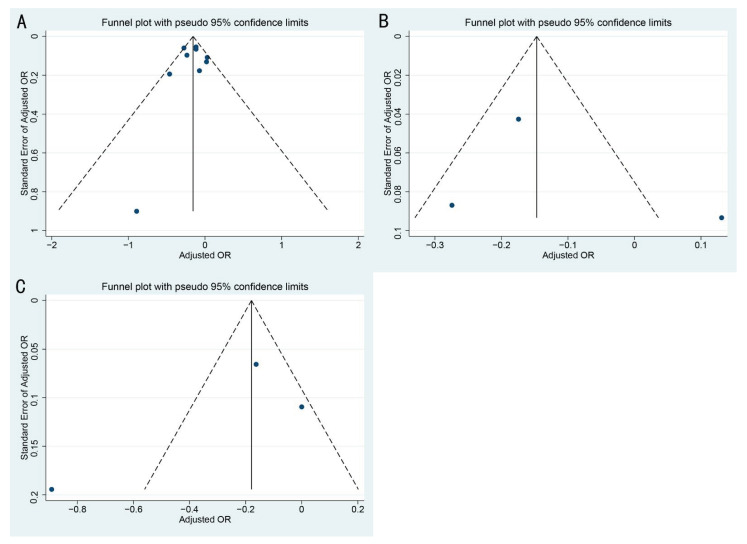
Funnel plots for the associations between influenza vaccination and SARS-CoV-2 infection and clinical outcomes: (**A**) adjusted OR of SARS-CoV-2 infection (**B**) mortality (**C**) hospitalization.

The summary of the publication bias are presented in Table 5 and Figure 4. Both funnel plots and Egger’s tests showed no evidence of publication bias for the pooled estimates and adjusted estimates of the association between influenza vaccination and SARS-CoV-2 infection (all *p* > 0.05). Neither evidence of publication bias for the association between influenza vaccination and COVID-19 related hospitalization or COVID-19-related mortality were observed (all *p* > 0.05).

## 4. Discussion

To our best knowledge, this is the first meta-analysis that systematically assessed the association between influenza vaccination and SARS-CoV-2 infection, and the association between influenza vaccination and clinical outcomes of SARS-CoV-2 infection. We ultimately included a total of 16 studies (12 on SARS-CoV-2 infection and six on clinical outcomes of SARS-CoV-2 infection, two on both) that had evaluated the associations. We observed a significant association between influenza vaccination and SARS-CoV-2 infection by pooling the adjusted estimates, while no evidence of the association was found between influenza vaccination and any of the clinical outcomes (hospitalization, mortality, and intensive care) among COVID-19 infected patients.

In the era of the COVID-19 pandemic, simply having licensed vaccines is not enough to achieve global control of COVID-19. Vaccines also need to be globally allocated and widely deployed [33]. Currently, only 4.3% of the global population have received at least one dose of a COVID-19 vaccine [34]. Vulnerable groups, such as the elderly and pregnant women, are usually not specifically included in COVID-19 clinical trials. Meanwhile, considerable mutation of SARS-CoV-2 has occurred since its initial emergence, which may have impact on the effectiveness of current vaccines and should be considered in vaccine design and development [35].

Influenza vaccination is regarded as the most effective measure to prevent influenza and influenza-related complications, especially for high-risk populations [36]. Previous studies suggested that older adults and pregnant women with influenza vaccination had a significantly lower risk of getting laboratory-confirmed influenza (older adults: risk ratio (RR) 0.76, 95%CI: 0.65–0.90; pregnant women: RR 0.3, 95% CI: 0.26–0.35) [37,38], which demonstrated relatively high effectiveness of influenza vaccines and highlighted the necessity of influenza vaccination. Restivo et al. [39] reported that influenza vaccine effectiveness was 39% (95%CI: 32–46%) for hospital visits and 57% (95%CI: 30–74%) for hospitalization among children, and the rate was 25% (95%CI: 6–40%) for visits and 14% (95%CI: 7–21%) for hospitalization among older adults. Cheng et al. [40] conducted a meta-analysis that included 29 studies and found that influenza vaccination was associated with a lower risk of overall adversary respiratory outcomes (including asthma, chronic obstructive pulmonary disease, unspecific respiratory diseases, respiratory failure, respiratory infections, pneumonia, and respiratory mortality) in the group aged over 65, from which significant associations were detected between influenza vaccination and pneumonia (aRR 0.79, 95 % CI: 0.65−0.95) and respiratory mortality (aRR 0.79, 95 % CI: 0.67−0.92). These results showed that influenza vaccination was effective at protecting vulnerable populations from influenza and its complications.

Due to the constant slight changes that influenza viruses undergo each season, influenza vaccines are required to be modified annually to maintain their effectiveness. Though influenza vaccination has been recommended by the WHO and other global organizations, annual influenza vaccine coverage remains to be relatively low in vulnerable populations. The overall influenza vaccination uptake rate among subjects with high-risk chronic conditions in Spain in 2017 was only 40.1% and decreased significantly from 2014 [41]. Though the rate among American children aged from 6 months to 17 years old increased from 16.70% during 2004/2005 to 49.43% during 2015/2016, it was still far below the U.S. Healthy People 2020 target of 70% annual influenza vaccination coverage among children [42]. In France, influenza vaccine coverage among pregnant women was only 7.4% in 2015/2016 season [43]. Effective interventions and tailored measures should be conducted to promote influenza vaccination and reduce vaccine hesitancy among high-risk populations.

Apart from playing an important role in influenza prevention, seasonal influenza vaccination is considered to have additional value in the strained period of COVID-19 pandemic. Influenza vaccination can reduce influenza-related hospital visits, thereby largely reducing the potential risk of respiratory infectious diseases, such as COVID-19, alleviating the burden of health care systems and saving medical resources for the treatment of other severe diseases [44]. It is also believed that being protected against influenza by vaccination enhances the accuracy of COVID-19 diagnosis and the specificity of COVID-19 surveillance [45].

In our study, we found that the influenza vaccination was associated with a lower risk of SARS-CoV-2 infection. One possible explanation was that those who received influenza vaccinations in the past seasons tended to pay more attention to their health status, thus they might have been more compliant with COVID-19 prevention measures, such as social distancing and wearing masks, which reduced their potential risk of infection. In addition, influenza vaccination could reduce the risk of influenza, which reduces the possibility of hospital visits and the risk of SARS-CoV-2 infection in high-risk areas, such as hospitals. Another possible theory that could explain influenza vaccine’s protective effect against COVID-19 is the trained immunity process. Influenza vaccines may induce non-specific activation of innate immune cells (e.g., natural killer cells) by increasing proinflammatory cytokine production, thereby triggering the non-specific protective effects against the diseases caused by heterologous viruses [46,47]. Such a mechanism has been demonstrated in Bacillus Calmette–Guérin (BCG) vaccine’s protective effective against malaria [48]. Accelerated natural killer cells and monocyte activation that correlated with reduced parasitemia was observed in the BCG vaccinated volunteers, which was consistent with the possibility of trained immunity. Likewise, influenza vaccination might be associated with a lower risk of COVID-19 via trained immunity. The underlying mechanism of the potential association between influenza vaccination and SARS-CoV-2 infection awaits further exploration.

Although we observed no evidence of a significant association between influenza vaccination and the lower risk of clinical outcomes among COVID-19 patients, Hui et al. [49] found that exposure of influenza A virus may upregulate the angiotensin-converting enzyme 2 (ACE2) receptors in alveolar epithelial cells, which facilitates SARS-CoV-2 virus to enter into alveolar epithelial cells and worsen clinical outcomes of SARS-CoV-2 infection. However, such effect was not observed in human macrophages. Bai et al. [3] found that influenza A virus promotes the infectivity of SARS-CoV-2 virus. The co-infection of influenza A virus and SARS-CoV-2 virus in mice resulted in increased SARS-CoV-2 viral load and more severe lung damage. Therefore, influenza vaccination may have a positive effect on better clinical outcomes of SARS-CoV-2 infection. More conclusive evidence is needed in the future.

In the subgroup analysis, statistics indicated that regions might be the potential sources of heterogeneity for the association between influenza vaccination and SARS-CoV-2 infection. The regional difference of the association between influenza vaccination and SARS-CoV-2 infection might result from various factors, including the diverse characteristics and immunity of the populations, the difference in the toxicity of SARS-CoV-2 viruses and dominant strains, and the efforts of COVID-19 prevention and control across regions.

The limitations in this study are listed as follows. First, subgroup analyses and sensitivity analyses on the association between influenza vaccination and the clinical outcomes of SARS-CoV-2 infection were not performed due to the limited number of studies. Similarly, publication bias was not adequately assessed. More relevant studies are needed to provide more precise pooled estimates. Second, only observational studies were available and included in our meta-analysis, so that the evidence regarding the intervention was not as strong as randomized controlled trials [50]. Nonetheless, this meta-analysis was performed strictly in accordance with the MOOSE guideline. Studies included in this meta-analysis took certain measures to minimize potential biases in selection and confounders, and all showed low to moderate risk of biases. Third, the exact influenza vaccination seasons and the confirmation of COVID-19 patients in some of the included studies were not specifically defined. Age groups and types of influenza viruses were not able to be used as grouping variables in subgroup analyses for lack of sufficient feasible studies with clear definitions and classifications. Additionally, studies included in the meta-analysis were all conducted before December 2020 when COVID-19 vaccines were not licensed yet. Some participants may have entered COVID-19 vaccine clinical trials and received vaccines, but were not reported in the studies, which may have impact on our analysis. However, the proportion of volunteers in COVID-19 vaccine trials could be very low and had little effects on our study. Last it was not possible to fully evaluate the possibility of overlapping participants among the included studies due to limited data. Nonetheless, this is the first meta-analysis that systematically assessed the association between influenza vaccination and COVID-19 and its outcomes, which could provide evidence-based medicine basis for the prevention of COVID-19 and influenza vaccination promotion.

## 5. Conclusions

This meta-analysis indicates that the influenza vaccination is associated with a lower risk of SARS-CoV-2 infection, while its association with clinical outcomes (mortality, hospitalization, and intensive care) of SARS-CoV-2 infection is not found. In the dual epidemics of the COVID-19 pandemic and influenza, it is especially crucial for policymakers to implement strategies aimed at promoting influenza vaccination, and that relevant public health campaigns and policy initiatives should be put in place to inform people of influenza vaccination benefits for the prevention and control of COVID-19. More evidence-based studies are urgently warranted to explore the association between influenza vaccination and SARS-CoV-2 infection and its outcomes, along with more in-depth research needed to further explain the underlying mechanisms for the associations.

## Figures and Tables

**Table 1:** Baseline characteristics of the 12 included studies that assessed the association between influenza vaccination and SARS-CoV-2 infection.

Study	Study Design	Vaccination Season	Identification of COVID-19	Country	Sample Size	Infected (n)/Vaccinated(n)	Infected (n)/Unvaccinated (n)	Adjusted Estimate (95%CI)	Quality Score and Risk of Bias Assessment	Adjusted Factors
Massoudi et al., (2021) [21]	Case-control study	2019–2020	pulmonologist-confirmed	Iran	261	3/90	77/171	-	6(moderate)	-
Kissling et al., (2021) [18]	Case-control study	2019–2020	rt-PCR	Europe ^a^	1701	68/429	157/1272	0.93 (0.66–1.32)	8 (low)	Study site, time, age, sex, and chronic condition
Ragni et al., (2020) [22]	Case-control study	2019–2020	rt-PCR	Italy	17,608	1676/5427	3209/12,181	0.89 (0.80–0.99)	9 (low)	Age, sex, Charlson index, and time of the swab test
Belingheri et al., (2020) [23]	Cross-sectional study	2019–2020	rt-PCR	Italy	3520	28/817	100/2703	0.41 (0.07–2.39)	7 (low)	Age, sex, and an interaction term between age and the vaccination intake in 2019/3020
Vila-Córcoles et al., (2020) [24]	Retrospective cohort study	2019–2020	rt-PCR	Spain	1547	189/705	160/842	0.63 (0.43–0.92) ^b^	8 (low)	Age, sex, and comorbidities
Pawlowski et al., (2021) [26]	Retrospective cohort study	2019–2020	rt-PCR	America	25,582	442/12,791	521/12,791	-	8 (low)	-
Jehi et al., (2020) [27]	Prospective cohort study	-^c^	rt-PCR	America	11,672	384/6324	434/5348	-	7 (low)	-
Vila-Córcoles et al., (2020) ^d^ [25]	Retrospective cohort study	2019–2020	rt-PCR	Spain	78,883	205/22,606	175/56,277	1.02 (0.79–1.32) ^b^	7 (low)	Age, sex, comorbidities, and medications use.
Martínez-Baz et al., (2020) ^e^ [16]	Prospective cohort study	2019–2020	rt-PCR	Spain	10,714	155/3677	248/7037	1.03 (0.83–1.27)	7 (low)	Age groups, sex, major chronic conditions, profession, and any ILI diagnosis in the previous five years
Noale et al., (2020) [28]	Cross-sectional study	2019–2020	rt-PCR	Italy	6680	562/2246	1114/4434	0.89 (0.78–1.01)	8 (low)	Age, sex, education, area of residence, self-reported comorbidities, and smoking status
Green et al., (2020) [29]	Cross-sectional study	2019–2020	rt-PCR	Israel	22,563	244/4711	1580/17,852	0.79 (0.67–0.98)	9 (low)	Age, ethnic, smoking status, socioeconomic status, and comorbidities
Conlon et al., (2021) [30]	Retrospective cohort study	2019–2020	rt-PCR	America	27,201	525/12,997	693/14,204	0.76 (0.68–0.86)	8 (low)	Ethnicity, race, sex, age, BMI, Elixhauser score, smoking status, and comorbidities

^a^: France, Netherlands, Sweden ^b^: aRR, the others are all aOR. ^c^: Not specifically defined, but records can be retrieved in the health care system. ^d^: the uninfected populations contain those who had suspected infection but were not tested. ^e^: the uninfected populations contain those who were not tested with no suspected infection. rt—PCR, reverse transcription-polymerase chain reaction.

**Table 2:** Baseline characteristics of the six included studies that assessed the association between influenza vaccination and SARS-CoV-2 outcomes.

Study	Study Design	Vaccination Season	Identification of COVID-19	Country	Sample Size	Events (n)/Vaccinated(n)	Events (n)/Unvaccinated (n)	Adjusted Estimate (95%CI)	Quality Score and Risk of Bias Assessment	Adjusted Factors
**Intensive Care**										
Pawlowski et al., (2020) [26]	Retrospective cohort study	2019–2020	rt-PCR	America	959	15/441	16/518	-	8 (low)	-
de la Cruz Conty et al., (2021) ^a^ [17]	Prospective cohort study	- ^b^	rt-PCR	Spain	1150	7/438	15/712	-	7 (low)	-
Fink et al., (2020) [10]	Retrospective cohort study	- ^b^	Clinical diagnosis^c^	Brazil	53,752	-	-	0.93 (0.87–0.99)	7 (low)	Age, sex, race, educational level, treatment facility, and comorbidities
Yang et al., (2021) [32]	Retrospective cohort study	2019–2020	rt-PCR	America	2005	3/214	133/1791	0.30 (0.07–0.85)	8 (low)	Age, sex race/ethnicity, hypertension, and comorbidities
**Hospitalization**										
Pawlowski et al., (2020) [26]	Retrospective cohort study	2019–2020	rt-PCR	America	959	74/441	78/518	-	8 (low)	-
Yang et al., (2021) [32]	Retrospective cohort study	2019–2020	rt-PCR	America	2005	43/214	747/1791	0.41 (0.28–0.60)	8 (low)	Age, sex race/ethnicity, hypertension, and comorbidities
Ragni et al., (2020) [22]	retrospective cohort study	2019–2020	rt-PCR	Italy	17,608	-	-	0.84 (0.83–1.29) ^d^	7 (low)	Age, sex, Charlson index, and time of the swab test
Wilcox et al., (2021) ^e^ [31]	retrospective cohort study	2019–2020	rt-PCR	England	6921	1166/2613	1584/4308	0.85 (0.75–0.97)	8 (low)	Age, sex, BMI, socioeconomic status, smoking status, frailty score, comorbidities, and the number of prescribed medications
**Mortality**										
Fink et al., (2020) [10]	Retrospective cohort study	- ^b^	Clinical diagnosis ^c^	Brazil	53,752	-	-	0.84 (0.77–0.91)	7 (low)	Age, sex, race, educational level, treatment facility, and comorbidities
Ragni et al., (2020) [22]	retrospective cohort study	2019–2020	rt-PCR	Italy	17,608	-	-	1.14 (0.95–1.37) ^d^	7 (low)	Age, sex, Charlson index, and time of the swab test
Wilcox et al., (2021) [31]	retrospective cohort study	2019–2020	rt-PCR	England	6921	372/2613	553/4308	0.76 (0.64–0.90)	8 (low)	Age, sex, BMI, socioeconomic status, smoking status, frailty score, comorbidities, and the number of prescribed medications

^a^: Intensive care unit admission/mechanical ventilation/septic shock. ^b^: Not specifically defined but records can be retrieved in the health care system. ^c^: 79.8% of these patients had a documented positive rt-PCR test. ^d^: aRR, the others are all aOR. ^e^: Hospitalization or all-cause mortality. rt—PCR, reverse transcription-polymerase chain reaction.

**Table 3:** Summary of the overall association between influenza vaccination and SARS-CoV-2 infection and clinical outcomes.

Outcomes	Number of Studies	*I*^2^ Value (%)	*p* Value	Adjusted Estimates ^a^ (95%CI)	
				Fixed Effects Model	Random Effects Model
SARS-CoV-2 infection	9	41.1	0.09	0.86 (0.81–0.91)	0.86 (0.79–0.94)
Intensive care	2	68.2	0.08	0.93 (0.87–0.99)	0.63 (0.22–1.81)
Hospitalization	3	87.6	<0.01	0.84 (0.75–0.93)	0.74 (0.51–1.06)
Mortality	3	82.5	<0.01	0.86 (0.81–0.93)	0.89 (0.73–1.09)

^a^: Adjusted OR or adjusted RR.

**Table 4:** Subgroup analyses of the association between influenza vaccination and SARS-CoV-2 infection.

Grouping Variables	No. of Studies	Random Effects Model			Fixed Effects Model		
		Adjusted Estimate (95%CI)	*I^2^* Value (%)	*p* Value	Adjusted Estimate (95%CI)	*I^2^* Value (%)	*p* Value
**Region**	9			0.04 ^a^			0.03 ^a^
Europe	7	0.91 (0.84–0.98)	10.4	<0.01 ^b^	0.90 (0.84–0.97)	10.4	<0.01 ^b^
Asia	1	0.79 (0.65–0.96)	-	-	0.79 (0.65–0.96)	-	-
America	1	0.76 (0.68–0.85)	-	-	0.76 (0.68–0.85)	-	-
**Sample size**	9			0.34 ^a^			0.06 ^a^
Sample size <20,000	6	0.89 (0.82–0.97)	13.3	0.33 ^b^	0.89 (0.83–0.96)	13.3	0.33 ^b^
Sample size ≥20,000	3	0.82 (0.71–0.96)	52.2	0.12 ^b^	0.80 (0.73–0.88)	52.2	0.12 ^b^
**Study design**	9			0.83 ^a^			0.55 ^a^
Case-control study	2	0.89 (0.81–0.99)	0.0	0.81 ^b^	0.89 (0.81–0.99)	0.0	0.81 ^b^
Cross-sectional study	3	0.85 (0.77–0.95)	0.0	0.43 ^b^	0.85 (0.77–0.95)	0.0	0.43 ^b^
Cohort study	4	0.86 (0.70–1.05)	71.7	0.01 ^b^	0.83 (0.75–0.91)	71.7	0.01 ^b^

^a^: *p* value for subgroup difference. ^b^: *p* value for heterogeneity.

**Table 5:** Summary of publication bias on the association between influenza vaccination and SARS-CoV-2 infection and clinical outcomes.

Outcomes	*t* Value	*p* Value
SARS-CoV-2 infection	−0.19	0.85
Mortality	0.46	0.73
Hospitalization	−0.87	0.55

Results from adjusted OR.

## Data Availability

Data are available from the corresponding author by request.

## Supplementary Materials

The following are available online at https://www.mdpi.com/article/10.3390/vaccines9050529/s1, Table S1: Summary of the overall association between influenza vaccination and SARS-CoV-2 infection and clinical outcomes by crude OR; Table S2: Subgroup analyses of the association between influenza vaccination and SARS-CoV-2 infection by crude OR; Figure S1: Forest plots for subgroup analysis on the association between influenza vaccination and SARS-CoV-2 infection by random effects model: (**A**) stratified by region (**B**) stratified by sample size (**C**) stratified by study design.
