# Supplementary material for: The Association between Influenza Vaccination and COVID-19 and Its Outcomes: A Systematic Review and Meta-Analysis of Observational Studies
Source: Vaccines (Basel). 2021 May 20;9(5):529. doi: 10.3390/vaccines9050529 (PMC8161076; doi:10.3390/vaccines9050529)
Supplement: Supplementary file 1 [file vaccines-09-00529-s001.zip › vaccines-1206565-supplementary.pdf]

Supplementary Table 1. Summary of the overall association between influenza vaccination and SARS-CoV-2 infection and clinical outcomes by crude OR

Outcomes	Number of studies	I^2 value (%)	P value	Crude OR (95%CI)	
				Fixed effect model	Random effect model
SARS-CoV-2 infection	9	41.1	0.09	1.00 (0.96-1.04)	0.99 (0.78-1.25)
Intensive care	2	68.2	0.08	0.55 (0.34-0.87)	0.57 (0.20-1.62)
Hospitalization	3	87.6	<0.01	1.21 (1.11-1.33)	0.83 (0.37-1.84)
Mortality	3	82.5	<0.01	1.13 (0.98-1.30)	1.13 (0.98-1.30)

Supplementary Table 2. Subgroup analyses of the association between influenza vaccination and SARS-CoV-2 infection by crude OR

Grouping variables	No. of studies	Crude OR (95%CI)	I^2 value (%)	P value	Crude OR (95%CI)	I^2 value (%)	P value
**Region**	12			<0.01[a]			<0.01[a]
Europe	7	1.27 (1.20-1.34)	93.2	<0.01[b]	1.37 (1.07-1.75)	93.2	<0.01[b]

Asia	2	0.52 (0.46-0.60)	94.5	<0.01[b]	0.16 (0.01-2.13)	94.5	<0.01[b]
America	3	0.80 (0.75-0.86)	13.1	0.32[b]	0.80 (0.74-0.87)	13.1	0.32[b]
**Sample size**	12			0.85 [a]			0.85 [a]
Sample size <10000	5	1.03 (0.94-1.14)	90.6	<0.01[b]	0.89 (0.58-1.36)	90.6	<0.01[b]
10000<=sample size<20000	3	1.13 (1.06-1.20)	95.4	<0.01[b]	1.03 (0.72-1.48)	95.4	<0.01[b]
Sample size >=20000	4	0.85 (0.80-0.91)	98.3	<0.01[b]	1.03 (0.60-1.77)	98.3	<0.01[b]
**Study design**				<0.01 [a]			0.23 [a]
Case-control study	3	1.21 (1.13-1.30)	93.7	<0.01[b]	0.64 (0.30-1.37)	93.7	<0.01[b]
Cross-sectional study	3	0.77 (0.71-1.84)	94.8	<0.01[b]	0.79 (0.51-1.24)	94.8	<0.01[b]
Cohort study	6	0.98 (0.93-1.05)	96.9	<0.01[b]	1.18 (0.81-1.71)	96.9	<0.01[b]

a: P value for subgroup difference.

b: P value for heterogeneity.

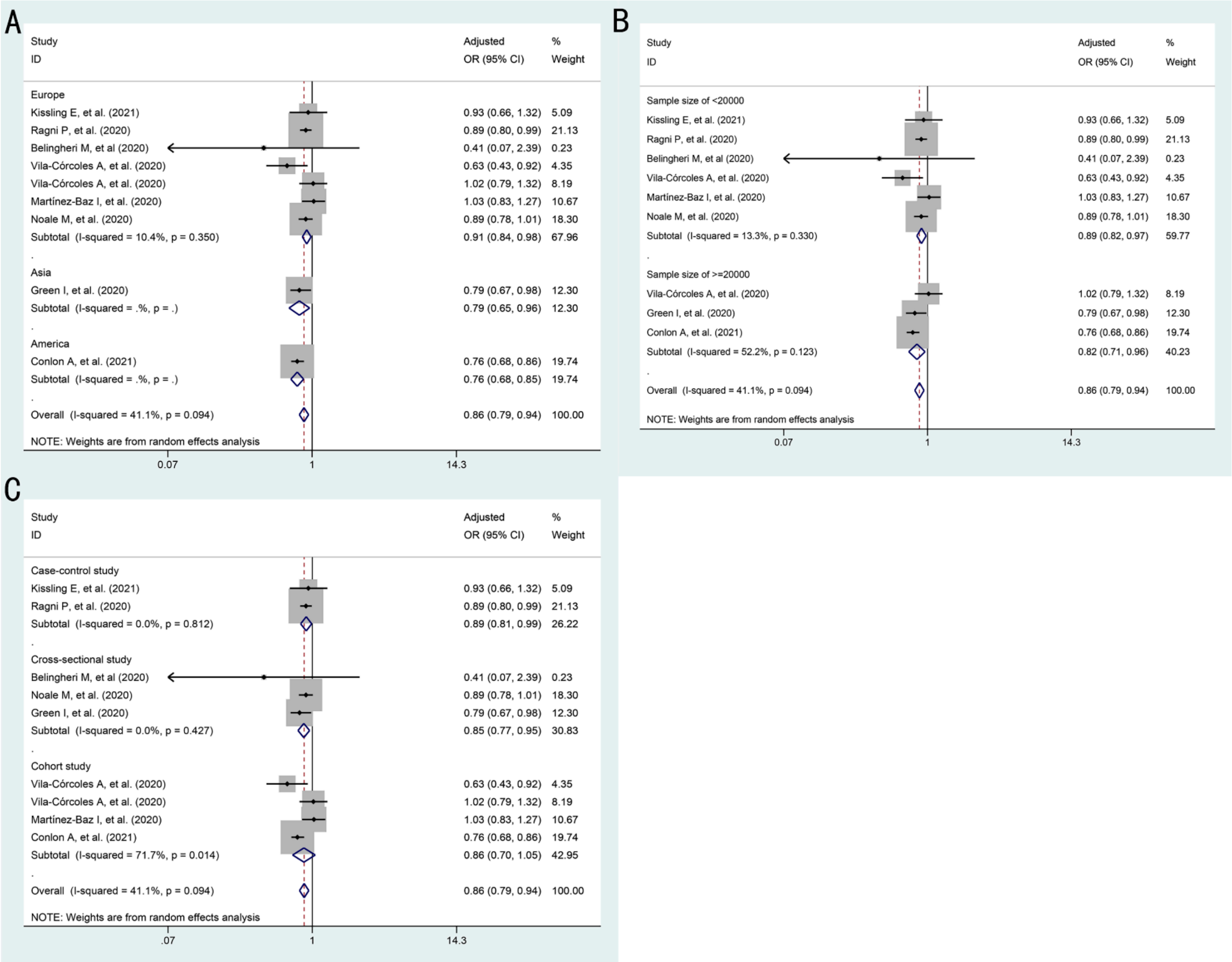
A
Study
ID
Adjusted
OR (95% CI)
%
Weight
Europe
Kissling E, et al. (2021) 0.93 (0.66, 1.32) 5.09
Ragni P, et al. (2020) 0.89 (0.80, 0.99) 21.13
Belingheri M, et al (2020) 0.41 (0.07, 2.39) 0.23
Vila-Córcoles A, et al. (2020) 0.63 (0.43, 0.92) 4.35
Vila-Córcoles A, et al. (2020) 1.02 (0.79, 1.32) 8.19
Martínez-Baz I, et al. (2020) 1.03 (0.83, 1.27) 10.67
Noale M, et al. (2020) 0.89 (0.78, 1.01) 18.30
Subtotal (I-squared = 10.4%, p = 0.350) 0.91 (0.84, 0.98) 67.96
Asia
Green I, et al. (2020) 0.79 (0.67, 0.98) 12.30
Subtotal (I-squared = .%, p = .) 0.79 (0.65, 0.96) 12.30
America
Conlon A, et al. (2021) 0.76 (0.68, 0.86) 19.74
Subtotal (I-squared = .%, p = .) 0.76 (0.68, 0.85) 19.74
Overall (I-squared = 41.1%, p = 0.094) 0.86 (0.79, 0.94) 100.00
NOTE: Weights are from random effects analysis
0.07
1
14.3
B
Study
ID
Adjusted
OR (95% CI)
%
Weight
Sample size of <20000
Kissling E, et al. (2021) 0.93 (0.66, 1.32) 5.09
Ragni P, et al. (2020) 0.89 (0.80, 0.99) 21.13
Belingheri M, et al (2020) 0.41 (0.07, 2.39) 0.23
Vila-Córcoles A, et al. (2020) 0.63 (0.43, 0.92) 4.35
Martínez-Baz I, et al. (2020) 1.03 (0.83, 1.27) 10.67
Noale M, et al. (2020) 0.89 (0.78, 1.01) 18.30
Subtotal (I-squared = 13.3%, p = 0.330) 0.89 (0.82, 0.97) 59.77
Sample size of >=20000
Vila-Córcoles A, et al. (2020) 1.02 (0.79, 1.32) 8.19
Green I, et al. (2020) 0.79 (0.67, 0.98) 12.30
Conlon A, et al. (2021) 0.76 (0.68, 0.86) 19.74
Subtotal (I-squared = 52.2%, p = 0.123) 0.82 (0.71, 0.96) 40.23
Overall (I-squared = 41.1%, p = 0.094) 0.86 (0.79, 0.94) 100.00
NOTE: Weights are from random effects analysis
0.07
1
14.3
C
Study
ID
Adjusted
OR (95% CI)
%
Weight
Case-control study
Kissling E, et al. (2021) 0.93 (0.66, 1.32) 5.09
Ragni P, et al. (2020) 0.89 (0.80, 0.99) 21.13
Subtotal (I-squared = 0.0%, p = 0.812) 0.89 (0.81, 0.99) 26.22
Cross-sectional study
Belingheri M, et al (2020) 0.41 (0.07, 2.39) 0.23
Noale M, et al. (2020) 0.89 (0.78, 1.01) 18.30
Green I, et al. (2020) 0.79 (0.67, 0.98) 12.30
Subtotal (I-squared = 0.0%, p = 0.427) 0.85 (0.77, 0.95) 30.83
Cohort study
Vila-Córcoles A, et al. (2020) 0.63 (0.43, 0.92) 4.35
Vila-Córcoles A, et al. (2020) 1.02 (0.79, 1.32) 8.19
Martínez-Baz I, et al. (2020) 1.03 (0.83, 1.27) 10.67
Conlon A, et al. (2021) 0.76 (0.68, 0.86) 19.74
Subtotal (I-squared = 71.7%, p = 0.014) 0.86 (0.70, 1.05) 42.95
Overall (I-squared = 41.1%, p = 0.094) 0.86 (0.79, 0.94) 100.00
NOTE: Weights are from random effects analysis
.07
1
14.3

Supplementary Figure 1: Forest plots for subgroup analysis on the association between influenza vaccination and SARS-CoV-2 infection by random effects model: (A) stratified by region (B) stratified by sample size (C) stratified by study design.
